# Boundaries Are Blurred: Wild Food Plant Knowledge Circulation across the Polish-Lithuanian-Belarusian Borderland

**DOI:** 10.3390/biology12040571

**Published:** 2023-04-09

**Authors:** Julia Prakofjewa, Matteo Sartori, Povilas Šarka, Raivo Kalle, Andrea Pieroni, Renata Sõukand

**Affiliations:** 1 Department of Environmental Sciences, Informatics and Statistics, Ca’ Foscari University of Venice, Via Torino 155, 30172 Venezia, Italy; 2 Department of History, University of Concepción, Edmundo Larenas 240, Concepción 4030000, Chile; 3 University of Gastronomic Sciences, Piazza Vittorio Emanuele II 9, 12042 Pollenzo, Italy; 4 Medical Analysis Department, Tishk International University, 100 Meter Street and Mosul Road, Erbil 44001, Iraq

**Keywords:** ethnobotany, wild food plants, local ecological knowledge, Poland, Lithuania, Belarus, cross-border, cross-cultural

## Abstract

**Simple Summary:**

Knowledge of plants and their uses is an essential link between people and the environment. To foster biocultural diversity as a vehicle for mutually beneficial coexistence, we need to understand how plant-related knowledge circulates. Considering the rapid loss of biocultural diversity, especially in peripheral areas, the local dimensions of ecological knowledge circulation merit greater scholarly attention. Exploring the current Polish-Lithuanian-Belarusian borderland, we found relatively homogeneous wild food plant knowledge circulated within historically united, yet now separated, local communities of Lithuanians and Poles. However, we call for deeper qualitative analysis in order to detect recent changes in the flow of knowledge.

**Abstract:**

The circulation of local ecological knowledge (LEK) is a promising avenue of research for wild plant studies. To encourage the acceptance, celebration, and appreciation of biocultural diversity, which is rapidly disappearing nowadays, we need to estimate and assess multifaceted local ecological knowledge. It has direct application for local communities in informing effective policies for improving food security and building community-specific responses to environmental and social transitions. The present study draws on data collected among two ethnic groups—Lithuanians and Poles—via 200 semi-structured in-depth interviews and participant observation conducted in 2018 and 2019 in Podlasie Voivodeship (Poland), the Vilnius Region (Lithuania), and the Hrodna Region (Belarus). We aimed to observe LEK circulation in the border area through cross-ethnic and cross-country comparisons. A total of 2812 detailed use reports of wild plants were recorded. In total, 72 wild plant taxa belonging to 33 plant families were used across the food domain. Our findings show that cross-country differences were minimal, while there was some variation between the ethnic groups selected as case studies. We emphasize the need, in future studies, to combine quantitative research with qualitative approaches in order to more thoroughly identify peculiarities of cross-border circulation as a reservoir for community food resilience and biocultural diversity.

## 1. Introduction

Biocultural diversity is rapidly disappearing [1,2], especially in peripheral areas [3]. To encourage the acceptance, celebration, and appreciation of biocultural diversity, we need to estimate and assess the importance of the local dimensions of ecological knowledge. 

Border regions have been at the center of scientific debate from different perspectives [4,5,6]. Driven by various political, social, and cultural processes, human activities such as border shifts strongly modify natural environments [7] and impact the flow of knowledge. Border areas might represent places to study environmental knowledge as a dynamic process [8]. Local communities constantly reshape their knowledge through interconnections, mutual influences, and non-linear flows of information [9,10,11]. Depending on boundary appearance/disappearance and opening/closure, the border area could be a barrier, filter, or contact zone [12] where knowledge can or cannot circulate.

The mixing of different cultural elements and a dynamically changing environment in border areas foster creating and maintaining multiple communication channels between local communities [13]. At the same time, in borderlands, intensive circulation of knowledge might contribute to the formation of shared uses and unique cultural realities that contradict the image of boundaries as a barrier [14]. The separations created by states provide clues to the development of unofficial social relations and hybrid manifestations, specifically, language confluence [15]. Several studies have highlighted the so-called “border paradox” [16] where national boundaries have determined and facilitated the creation of shared cross-border flows of knowledge. Borders, as the social construction of peripherality [17], might lead to the homogenization of knowledge [18].

Recently, there has been a growing body of research concerned with the importance of borders in LEK transmission [19,20]. Several researchers have noted changes in the use of natural resources [21] (p. 60) and significant divergence in LEK between the divided border communities [22,23], despite many years of living together in the same area and sharing the same religious faith [24], as well as accessing multilingual folk and scientific literature regarding the use of wild plants [25]. In this vein, various ethnobotanical border studies have found that differences within the compared ethnic groups are more pronounced than those with other local communities currently inhabiting the same country [26,27].

Nevertheless, peripheral border areas represent an ideal study site for exploring the phenomenon of LEK circulation in its temporal and spatial dynamics. The present-day Polish-Lithuanian-Belarusian triangle has been subjected to a series of border shifts. For centuries, the triangle between Poland, Lithuania, and Belarus has been a multi-linguistic, multi-religious, and multi-cultural area to a certain extent [28,29]. Historical conditions and its geographically peripheral location have made this region a place of cohabitation of various ethnic groups: Poles, Lithuanians, Belarusians, Ukrainians, Jews, Russians (predominantly Old-Believers), Tatars, Roma, etc. The studied cultural landscape has become a reservoir, and even a hotspot, of biological and cultural diversity. The selected area and its communities have been investigated from historical [30,31,32,33,34,35] and modern ethnobotanical perspectives [36,37,38,39]. Thus far, however, our understanding of the degree to which border shifts may result in the homogeneity of culturally unique knowledge has been limited.

The aims of the study were to (1) document LEK on wild food plants among Lithuanian and Polish communities in the Polish-Lithuanian-Belarusian borderland, (2) conduct cross-border and cross-ethnic comparisons in order to understand the dynamics of knowledge circulation within the region, and (3) evaluate the effects of border shifts on LEK circulation within the tri-border area. On the basis of the potential influence of state frontiers, we expect to see, as a general trend, knowledge heterogeneity among the three countries and relative knowledge homogeneity within the cross-border ethnic groups.

## 2. Materials and Methods

### 2.1. Study Site and Data Collection

**Figure 1:**
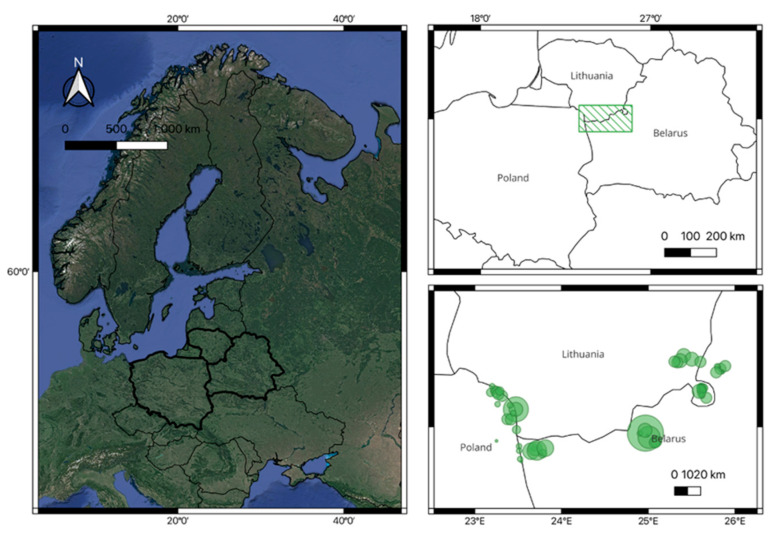
Map of the study area. The border region, which consists entirely of lowlands with a maximum altitude of ca. 240 m above sea level, extends over parts of northern Podlasie (NE Poland), Dzūkija (SE Lithuania), and various districts in the Neman River Basin (NE Belarus); designed with QGIS 3.22.16 ‘Białowieża’.

The data was collected, over six months in 2018 and 2019, via semi-structured interviews and participant observation conducted in 60 rural settlements in the regions of Podlasie Voivodeship (Poland), Vilnius (Lithuania), and Hrodna (Belarus). Most of the territory of the tri-border area is inhabited by people who nowadays self-identify as Polish [40,41,42]. Nevertheless, the selection of villages for fieldwork was also predetermined by the dispersed and compact settlements of Lithuanians in the study area (Figure 1).

**Figure 2:**
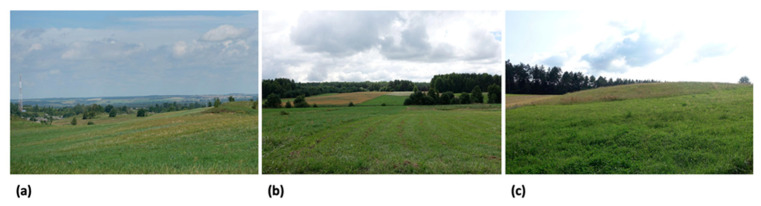
Typical landscapes of the (**a**) Belarusian, (**b**) Lithuanian, and (**c**) Polish parts of the studied border area. Credit: J.P., 2018–2019.

The studied tri-border area is located in the East European Platform and is characterized by considerable landform diversity, significant forest cover, and valuable geomorphologic features formed by continental glaciation [43]. The border region contains diverse ecosystems, such as abundant forests, meadows, wetlands, and waterbodies. Small patchwork fields and adjacent areas of arable land planted with various crops are characteristic features of the rural landscape of the study area (Figure 2). The study region’s soil is accorded little agricultural value, which justifies the introduction of afforestation schemes [44]. For the study sample, we mainly selected rural settlements close to forest ecosystems in all three case studies [45]. 

The local residents are mainly bi- or multilingual [46,47]. Our interviewees predominantly used more than one language/local dialect in communication (primarily Polish, Lithuanian, Belarusian, and Russian). Elderly interviewees from Belarus and Lithuania often declared that they speak (or their parents spoke) “pa prostu” or “pa tutejšamu” (which means ‘straightforward, easy, unsophisticated speech’, an uncodified vernacular form of Belarusian) [48,49]. Several times, our interviewees showed fluidity in their ethnic identity. For instance, in Lithuania, some people of Polish descent considered themselves both Poles and Lithuanians. In Belarus, older people identified themselves as Poles, while the younger generation declared themselves Belarusians. The strongest identification in all the surveyed groups was among Lithuanians.

The political landscape of the studied area was highly dynamic. Poland, Lithuania, and Belarus shared significant historical events from the 14th century through the middle of the 20th century [50]. Before 1939, all the territory of the study area was incorporated into the Second Polish Republic (with administrative borders between investigated settlements). Then, after Soviet invasion, the study region was divided among the Byelorussian Soviet Socialist Republic, the Lithuanian Soviet Socialist Republic, and the Polish People’s Republic. Therefore, there was a soft border between Belarus and Lithuania as they were both part of the Soviet Union, but they had a hard border with Poland. After the collapse of the USSR, between 1989 and 1992, Poland, Lithuania, and Belarus all gained independence. Finally, in 2004, Lithuania and Poland became members of the European Union, thus establishing a hard border with Belarus.

This research was carried out within the framework of an ethnobotanical study focusing on border regions of Eastern Europe (ERC Starting Grant no. 714874). In the interest of the umbrella project, our goal was to obtain a sample conforming to specific criteria: individuals approximately 40 years of age or more, representing both men and women, and belonging to ethnic groups (Polish and Lithuanian) living in all three researched countries. We included only local (born in the region and lived there for at least the last 30 years) rural residents. We used a pseudo-random sampling method, complementing it with occasional snowball sampling. To obtain more detailed information, we interviewed people in their homes or/and during walks in the surrounding area, which lasted from 30 min to 3 h, depending on the availability of the individual.

The study sample included a total of 200 people: 156 women and 44 men. We conducted interviews with 95 Lithuanians and 105 Poles, with an average age of 68.54 and 72.07 years, respectively. The discrepancy in gender arose because of the low number of elderly men in the study area. The majority of interviewees in the study sample were retired and had either worked on collective farms (in Belarus and Lithuania) or were small-scale farmers (in Poland). About 25% of the sample represents (former) teachers, librarians, and nurses from all three countries. All interviewees self-identified as Roman Catholic.

To evaluate the wild food plant LEK, the data was grouped by country and ethnic group. In total, we defined 6 case studies for comparison: (1) Lithuanians living in Belarus (BYLT), (2) Poles from Belarus (BYPL), (3) Lithuanians from Lithuania (LTLT), (4) Poles living in Lithuania (LTPL), (5) Lithuanians from Poland (PLLT), and (6) Poles living in Poland (PLPL). Furthermore, in every case study, we collected data on control variables that may affect the distribution of WFP knowledge within an ethnic group living in a specific country. These variables included: gender (0—female, 1—male), education (according to ISCED-11 [51] classification: 0—no schooling; 1—primary education; 2—lower secondary education; 3—upper secondary education; 4—post-secondary non-tertiary; and 5—equivalent tertiary education level), age (in years), and language (according to the number of declared languages spoken by an interviewee: 1–4, among which were Polish, Lithuanian, Belarusian, and Russian).

**Figure 3:**
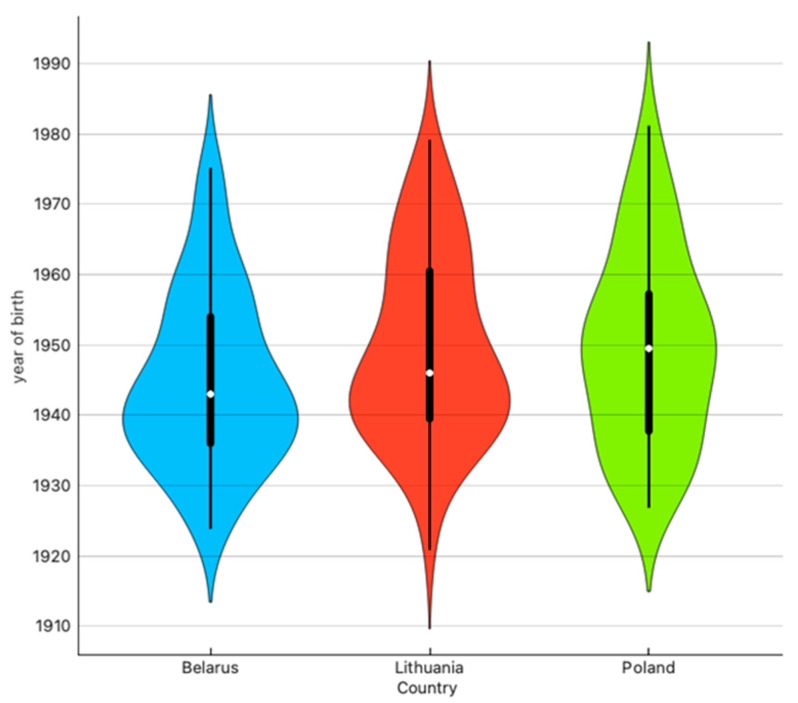
Violin plot of the age distribution of the sample in the three studied countries.

Table 1 shows the socio-demographic distribution of the sample selected for analysis. We found no statistically significant association between the interviewees’ ages among the six case studies (*p* = 0.099). Consequently, our cross-border study sample was relatively homogeneous by age (Figure 3).

The Code of Ethics of the International Society of Ethnobiology [52] was strictly followed. The research protocol was approved by the Ethics Committee of Ca’ Foscari University of Venice. Written and oral consent were obtained from all participants prior to the interviews. All interview recordings were subsequently transcribed, maintaining the linguistic and metacommunicative nuances for more transparency in and reproducibility of the statistical analysis.

Voucher specimens were collected for the wild taxa, when available, and subsequently deposited at the herbarium of Ca’ Foscari University of Venice (UVV): Lithuanian specimens bear accession numbers DZULT01–DZULT136 and DDZULT01–DDZULT42, and Polish specimens bear accession numbers DZUPL001–DZUPL107 and DDZUPL01–DDZUPL39. The total number of specimens collected was 324. Taxonomic identification, botanical nomenclature, and family assignments followed the Flora Europaea [53] and the Plants of the World Online database [54]. Local plant names were transliterated following the rules of the standard Belarusian and Russian languages.

### 2.2. Data Analysis

The information gathered from the interviewees was entered into a Microsoft Excel spreadsheet in the form of detailed use reports (DUR), where each interviewee mentioned the use of wild species and their preparation [55]. To explore knowledge circulation within the tri-border area, we conducted bivariate and multivariate analyses.

To test the homogeneity of the sample, we calculated cross-country differences based on the number of taxa used by a person and grouped the results by area, gender, age, education, and language spoken. We used Student’s *t*-test (for two variables) and ANOVA and chi-square test (for three or more variables) to determine whether differences in the number of plants mentioned were statistically significant. The statistical confidence level was set at *p* ≤ 0.05. We used Pearson’s correlation coefficient to test the relationship between the individual scores for the knowledge domains.

To conduct cross-ethnic and cross-country comparisons, Jaccard similarity indices were calculated following González-Tejero et al. [56]: $JI=\frac{C}{A+B-C}*100$, where *A* is the number of species/genera in sample *A*, *B* is the number of species/genera in sample *B*, and *C* is the number of species/genera common to *A* and *B*.

To perform the quantitative assessment of the collected data, we used the ethnobotanyR package [57]. Specifically, to evaluate the significance of wild food species for the studied local communities, several quantitative calculations were made. We quantified use reports and number of uses per species [58], fidelity level (FL) of the various uses of species [59], relative frequency of citation index (RFC) [60], cultural importance index (CI) [59], and informant consensus factor (ICF) [61]. The combination of these calculations offered a comprehensive evaluation of the importance of plants for the studied local communities (see Appendix A).

Statistical analysis and graph plotting was performed with Microsoft Excel (Data Analysis) and R-4.2.2 software(R Development Core Team; Venice, Italy) using various CRAN packages [62].

## 3. Results

We recorded the food uses of 72 wild plant taxa belonging to 33 plant families, the most representative of which were Asteraceae (10 taxa), Rosaceae (8 taxa), Ericaceae (6 taxa), and Lamiaceae (6 taxa) (Table 2). The collected data was divided into 2812 DUR, covering both current and past uses.

The most multifunctional taxa in all three regions (countries) of the studied border area were *Rubus idaeus* (used in 9 emic categories), used mainly for jam, non-alcoholic drinks, and snacks; *Vaccinium oxycoccos* (8), used as a seasoning, a snack, and for jam making; *Vaccinium vitis-idaea* (8), used primarily for jam, as a snack, and for recreational tea; and *Vaccinium myrtillus* (7), used mainly for jam, as a snack, and for non-alcoholic drinks. The most popular used taxa among all interviewees were *Rumex acetosa* (280 DUR), followed by *Vaccinium myrtillus* (268 DUR), *Armoracia rusticana* (223 DUR), *Betula* spp. (188 DUR), and *Carum carvi* (181 DUR).

The most popular food categories included soup made from *Rumex acetosa* (274 DUR), relish (seasoning) made from *Armoracia rusticana* (215), sap from *Betula* spp. (169), seasoning made from *Carum carvi* (138), and jam made from *Vaccinium myrtillus* (133). The most diverse emic food categories used within the three regions of the study area were recreational tea (40 plant taxa), snacks (mainly berries) (27), various additives (20), and non-alcoholic drinks (19).

**Figure 4:**
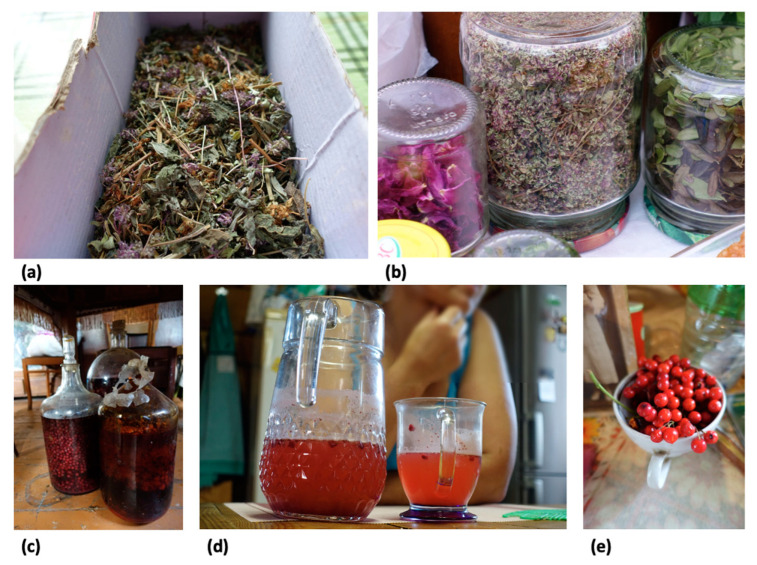
Wild species used for food: (**a**) mixed herbs dried for recreational tea, Lithuania; (**b**) herbs prepared for recreational tea, Poland; (**c**) wine made from *Rubus idaeus*, Belarus; (**d**) compote, made from *Fragaria vesca*, Lithuania; and (**e**) snack of *Viburnum opulus*, Belarus. Credit: J.P., 2018–2019.

The highest (100.00%) fidelity level in all three countries was found for the use of *Achillea millefolium*, *Artemisia vulgaris*, *Capsella bursa-pastoris*, *Equisetum pratense*, *Helichrysum arenarium*, *Hypericum* spp., *Leonurus cardiaca*, *Matricaria chamomilla*, *Melissa officinalis*, *Nepeta cataria*, *Thymus* spp., and *Tussilago farfara* for recreational tea (Figure 4); *Cirsium oleraceum*, *Heracleum sphondylium*, *Rumex acetosa*, and *Urtica urens* for soup; *Borago officinalis* and *Symphytum officinale* in salad; *Campanula* sp., *Corylus avellana*, and *Oenothera biennis* as a snack; *Armoracia rusticana*, *Origanum vulgare*, and *Thlaspi arvense* as a seasoning; *Aesculus hippocastanum* and *Cichorium intybus* as a food substitute; and *Alnus* spp. for smoking meat.

### 3.1. Sample Analysis

**Figure 5:**
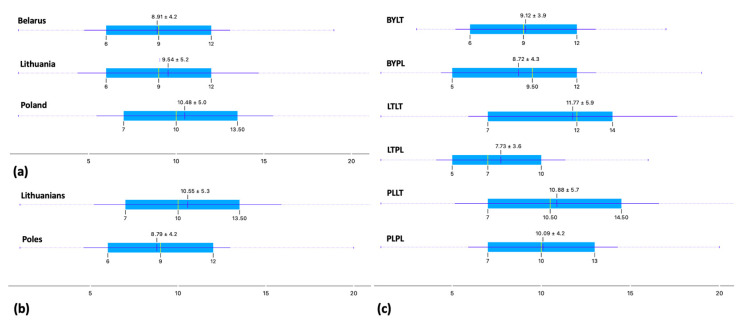
Box plots for the ‘number of taxa mentioned’ grouped by (**a**) country, (**b**) ethnic group, and (**c**) case study.

According to the use of wild plants for food, we detected no significant difference on the country level. However, we found a lower average score for plant species mentioned by Poles (mean 8.79) compared to Lithuanians (mean 10.55) (*p* = 0.011) and significant differences in wild food plants mentioned when comparing all six case studies among each other (*p* = 0.007) (Figure 5).

There was a significant difference in the number of plants used by the two genders, in which men reported using fewer plants than women (7.9 and 10.12 on average, respectively) (*p* = 0.007). We did not find statistically significant evidence of the impact of educational level (*p* = 0.331), nor the number of languages spoken (*p* = 0.495), on the number of used plants (Table 3).

**Figure 6:**
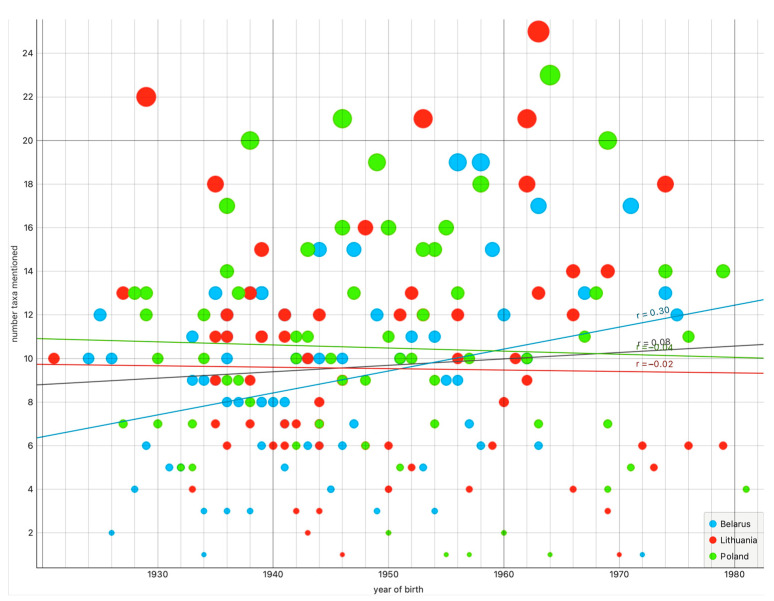
Distribution of the number of taxa mentioned in Belarus, Lithuania, and Poland according to the year of birth of interviewees. The size of the circle correlates with the number of taxa mentioned.

We observed that in all our cross-border case studies age did not play a significant role in the distribution of LEK (ANOVA: 1.883, *p* = 0.099). More than 20 taxonomic species were mentioned mostly by middle-aged adults. Pearson’s correlation coefficient between the age of interviewees and the plant species mentioned was negative (r = −0.076) and reflected a non-significant association (Figure 6).

### 3.2. Cross-Country and Cross-Ethnic Comparisons

**Figure 7:**
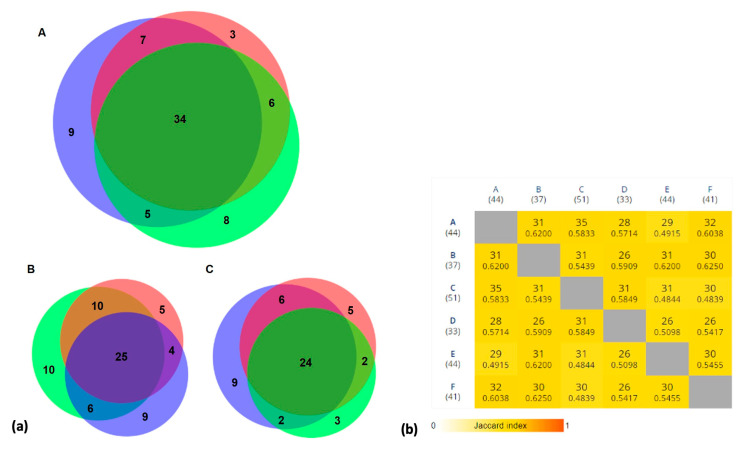
(**a**) Venn diagrams for the division of used taxa and use instances: A, recorded in Belarus (red), Lithuania (green), and Poland (violet); B, among Lithuanians living in Belarus (red), Lithuania (green), and Poland (violet); C, among Poles living in Belarus (red), Lithuania (green), and Poland (violet); (**b**) Jaccard similarity indices for the various compared groups based on detailed use reports, where A—Lithuanians in Belarus, B—Poles in Belarus, C—Lithuanians in Lithuania, D—Poles in Lithuania, E—Lithuanians in Poland, and F—Poles in Poland.

We found a high level of homogeneity (similarity) among the case studies with a core of 21 common taxa. Lithuanians from Lithuania used a greater diversity of taxa (52), whereas Poles from Lithuania (33) used fewer taxa but with greater intensity (based on DUR) (Figure 7).

The least amount of overlap in the gathered data, and thus the lowest Jaccard index (similarity coefficient) value, was between Lithuanians living in Lithuania and Poles (0.4839) and Lithuanians (0.4844) from Poland. A greater level of overlap in the use of wild plant taxa for food, and consequently a higher level of LEK homogenization, was observed between Poles living in Belarus and Lithuanians (0.6200) and Poles (0.6250) from Poland.

Little difference was found between ethnic groups and groups living in the same country. In this respect, the boundaries between ethnic groups are rather blurred, as they share 30 or 31 taxa.

The relative frequency of citation ranged between 0.826 and 0.014 in all three case studies (Table 4). Thus, we did not identify quantitative differences on the taxon level.

*Vaccinium myrtillus* (1.180) was the most culturally significant plant in all six case studies. It has a CI index value ranging between 1.343 (for Poles from Belarus) and 0.938 (for Lithuanians living in Poland). The next most culturally significant taxon was *Rubus idaeus* (0.815), with a range between 1.062 (for Lithuanians living in Poland) and 0.594 (for Poles from Poland), followed by *Rumex acetosa* with a CI index value of 0.795. For this latter taxon, the difference between studied cases was relatively low and ranged between 0.941 for Lithuanians from Belarus and 0.500 for Poles living in Lithuania. Interestingly, *Carum carvi* has a CI index value of 0.755, with greater cultural importance for Poles (0.938) in all three countries in comparison with Lithuanians (0.806). *Urtica dioica* is culturally significant in the studied communities and has a CI index value of 0.680, with a range between 1.156 for Lithuanians from Poland and 0.344 for Poles from Poland. Therefore, the results confirmed relative homogeneity among CI values obtained in the different cross-border case studies. The top ten species of wild food plants with the highest CI values were mentioned in every case study (see Appendix B).

The informant consensus factor (ICF) for the whole study border area was very high (0.970) (Table 5). A similar pattern was observed when considering the countries of Belarus (0.935), Lithuania (0.934), and Poland (0.933) separately, and when comparing the two ethnic groups: Lithuanians (0.959) and Poles (0.949).

## 4. Discussion

Nowadays, the Polish-Lithuanian-Belarusian borderland represents the result of many layers of past environmental processes and human interventions. We observed that wild food plant knowledge was relatively evenly distributed across the area regardless of the existing state boundaries, as we did not find statistically significant differences between countries. A high ICF value indicates an extraordinary level of agreement among interviewees in the whole studied region on the taxonomic level of wild plants used for food. Previously, a high ICF in the food domain was observed primarily in non-border areas [20,63,64].

Relatively homogeneous knowledge on the use of wild plants for food in the studied region might be explained by the fact that Poles and Lithuanians have resided in the investigated territories for centuries [50]. The flexible qualities of identity and the possession of different languages in the studied region facilitated the cross-border flow of knowledge, not only by creating shared connections between individuals but also by allowing bridge-building among other ethnic groups. Interestingly, no clear national identity as “tutejszy” (“from here”) has been observed for the rural population in this historical region, even in the interwar period [29]. Furthermore, the recorded fluid and floating identity in the border zone facilitated knowledge circulation.

We observed that nowadays the two relatively distinct studied groups still use, in everyday communication, “język tutejszy”/“mowa prosta” (local language) as a lingua franca. This certainly facilitated inter-ethnic communication in the multicultural border region and promoted the more open exchange of information. In certain cases, two local communities used to speak Russian. For a former Soviet territory, it is quite a widespread practice of inter-ethnic communication [65], especially considering that older and middle-aged respondents predominantly studied Russian at school.

Our field results indicated that for all three studied countries, the environment has changed and many plant species have disappeared. For instance, extinct plants included those that were used for recreational tea (*Centaurea cyanus* and *Nepeta cataria*) and as a snack (*Vaccinium uliginosum, Corylus avellana, Oxalis actosella*, and *Pinus sylvestris*). In addition, some taxa were used for food practices no longer in circulation: bread making (*Acorus calamus*), meat smoking (*Juniperus communis* and *Populus tremula*), and famine foods associated with WWII and the post-war period (*Stellaria media, Chenopodium album*, and *Heracleum sphondylium*). Some interviewees also stressed that plants such as *Armoracia rusticana* and *Carum carvi* have become feral and no longer need to be planted as they grow on their own, without intervention. Moreover, our field materials revealed that wild apple and pear trees have gone out of use, as they have been replaced by cultivated ones (*Malus sylvestris* and *Pyrus pyraster*).

Despite their extinction from the natural landscape, we found that many plants continue to exist in the discourse of an ethnic group, as they are still highly involved in food traditions (e.g., *Papaver* for making Christmas and Easter pastries, *Vaccinium oxycoccos* for making kissel, etc.). These traditions remain very strong in the studied communities as almost every interviewee noted that they try to keep making certain dishes within the family on major Catholic holidays so that now they buy all the ingredients in shops. Furthermore, we observe here an essential feature: even if the plant has fallen out of natural circulation due to the social and ecological changes that took place during the 20th century, it remains culturally important.

The homogeneity in LEK observed between Lithuanian and Polish communities settled in both Belarus and Lithuania may likely be explained by their long period of coexistence within the same (Soviet) social and political system, as already discussed in other post-Soviet ethnobotanical case studies [22,26]. The high homogeneity of wild food plant knowledge between Poles from Belarus and Poles from Poland may be the result of the long-term effects of a shared, common history before 1939 (actively emphasized by interviewees) and the current unrestricted communication between the two groups where the research was conducted, owing to a simplified border crossing system.

The identified convergent trajectories of LEK circulation among the studied ethnic groups may represent the primary response to recent cultural globalization forces. Globalization acts to foster relationships between heterogeneous communities, often transcending national borders, even though the flow of knowledge within national boundaries may be limited as well [66]. Thus, we cannot exclude the effects of globalization [67] on the blurring of borders and the statistically insignificant differences in plant taxa used nowadays by the studied ethnic groups. While powerful global forces such as market expansion and linguistic colonization may have a widespread erosional effect, this is not inevitable, and culture- and site-specific factors also determine the outcome [68,69,70].

Although the prevalence of high consensus levels for wild food species between Polish and Lithuanian interviewees living in Poland, Lithuania, and Belarus is significant, there are many levels of divergence in ethnobotanical knowledge noted between these two ethnic groups within the country case studies. Distinct cultural groups tend to diverge in food practices through specific cultural associations with consumable resources [63]. In particular, ethnic group-level statistically significant heterogeneity is observed within one country. The marked heterogeneity in LEK observed between Poles from Poland and Lithuanians from Lithuania can likely be explained by the presence of the hard Lithuanian-Polish border and the almost total lack of contact between the two communities during Communist/Soviet times. The closed Soviet-Polish border strongly influenced wild food plant knowledge circulation as, in all cases, Poland is quantitatively different from former Soviet Lithuania and Belarus. Free circulation of social discourse on wild food plants and free practical application (access to resources) are the basis for resistance and help develop adaptive food security strategies that allow substantially independent policy decisions.

## 5. Conclusions

We documented a high diversity of wild plants used for food within the studied cross-border region, while the number of plants used by each specific research group was considerably smaller. This and the high ICF obtained for the whole region show that every studied group has preserved (obtained) a fraction of the general wild food plant knowledge circulating within the region. This may signal the existence of long-term effects of common, shared traditional ecological knowledge within the entire region.

Our findings suggest that the divergences observed are possibly linked to various environmental, cultural, social, political, and economic shifts experienced by the studied countries. We also noticed clear differences on the discourse level, which would require separate qualitative analyses of attitudes and sentiments, which cannot be reflected in descriptive statistics. Our findings indicate that different permeabilities of former boundaries of the Soviet Union might have influenced wild food plant knowledge circulation (Belarusian-Polish vs Polish-Lithuanian borders). The consequences of various political settings on knowledge circulation needs to be addressed by future studies.

## Figures and Tables

**Table 1:** Sample distribution by gender, age, education, and language.

Variable	BYLT (*n* = 33)	BYPL (*n* = 36)	LTLT (*n* = 30)	LTPL (*n* = 37)	PLLT (*n* = 32)	PLPL (*n* = 32)
**Gender**						
0 Female	31	29	23	27	25	21
1 Male	2	7	7	10	7	11
**Age (years)**						
**Min/max**	min = 44 max = 89	min = 43 max = 94	min = 42 max = 89	min = 39 max = 97	min = 38 max = 90	min = 40 max = 92
Mean/dispersion	Mean 72.27 Dispersion 0.15	Mean 73.83 Dispersion 0.18	Mean 65.20 Dispersion 0.20	Mean 71.27 Dispersion 0.18	Mean 67.81 Dispersion 0.18	Mean 71.0 Dispersion 0.21
Standard deviation	11.029	13.534	13.299	12.650	12.400	15.151
**Education**						
0 no schooling	2	0	0	0	0	0
1 primary	12	15	7	12	5	8
2 lower secondary	6	8	4	6	11	6
3 upper secondary	1	4	5	7	7	4
4 post-secondary non-tertiary	7	7	13	12	5	13
5 equivalent tertiary education level	5	2	1	0	4	1
**Number of spoken languages**						
1	1	4	2	11	0	29
2	19	22	4	21	10	3
3	10	10	19	3	22	0
4	3	0	5	2	0	0
Mean	2.45	2.167	2.9	1.89	2.688	1.093

**Table 2:** Use of wild plants for food among Lithuanians living in Belarus (BYLT), Lithuania (LTLT), and Poland (PLLT); and Poles from Belarus (BYPL), Lithuania (LTPL), and Poland (PLPL). Local name (s): PL—mentioned among the Polish community; LT—among the Lithuanian community.

Family	Latin Name; Voucher Number	Local Name(s):	Used Part(s)	Preparation	Food Use	BYLT	BYPL	LTLT	LTPL	PLLT	PLPL
Acoraceae	*Acorus calamus* L.; DZUPL003, DZULT080	PL: aer, ajeras, aleras, jagier, kalmus, tatarak, ajer, air LT: ajerai, arieliai, ajeras, aleras, alerai, ajyr, areliai, tatarak, ajer	leaves	dried	under bread during baking	2	3	11	1	9	
				fresh	seasoning for bread						1
					under bread during baking	6		3	5	6	19
			stems	fresh	dessert					1	
					snack			1		2	
Amaranthaceae	*Chenopodium album* L.; DZULT105	PL: lebioda, lebeda, lebiada LT: balanda, balandos, lebeda	aerial parts	cooked	soup		9	1		5	
				dried	bread additive			1			
				fresh	salad			1		1	
			leaves	cooked	soup					1	
				dried	soup		1				
			young plants	cooked	soup					2	
Apiaceae	*Aegopodium podagraria* L.	PL: podagrycznik, śnitka, snyć LT: garšva	leaves	cooked	soup	1	2			1	
				dried	soup					1	
				fresh	salad		1				2
				frozen	soup					1	
			young leaves	fresh	salad			2			
	*Anethum graveolens* L.; DDZULT29, DZULT063	PL: koperek, krop, ukrop LT: krapus, krop, krapai, ukrop	aerial parts	fresh	seasoning for lactofermented cucumbers		1				
			seeds	dried	seasoning for bread	2					2
					seasoning for sauerkraut	5	4				
	*Carum carvi* L.; DDZULT40, DDZUPL36	PL: kmin, kminek, kmynai, kmynas, tmin, kmien LT: kmyn, kmynai, kmynas, kmin, tmin	seeds	dried	recreational tea			11	1	23	3
					seasoning	11	6	14	8	2	10
					seasoning for bread	2	2	2	4	12	9
					seasoning for cheese						1
					seasoning for lactofermented cucumbers				1		
					seasoning for meat	1	1		2		
					seasoning for sauerkraut	9	8	3	11	7	8
					seasoning for sausages	1	2				
					taste additive to alcohol	1		3		1	
				frozen	seasoning	1					
	*Heracleum sphondylium* L.	PL: boršč LT: barščiai, grobūzdai, barštis	leaves	cooked	soup	2		6	2		
				dried	soup			2			
				salted	soup			1			
Asteraceae	*Achillea millefolium* L.; DZUPL042, DDZUPL17, DZULT027, DZULT038, DZULT064	PL: kraujažolė, tysiačalistnik LT: kraujažolė	aerial parts	dried	recreational tea			3	2	3	
	*Arctium tomentosum* Mill.	PL: łopian	leaves	fresh	to preserve fresh meat						1
					salad						1
	*Artemisia vulgaris* L.; DZUPL040, DZUPL094, DZULT079	LT: kietis	aerial parts	dried	recreational tea			1			
	*Centaurea cyanus* L.; DDZULT31, DZULT110	PL: chaber LT: rugiagėlė, vosilkė, vosilkės, rugių gėlės, vosilkos	flowers	dried	recreational tea	1		4			1
				fresh	dessert	1					
	*Cichorium intybus* L.; DZUPL029, DZUPL075	PL: cykoria, cykoryj	roots	roasted	coffee substitute				1		1
	*Cirsium oleraceum* (L.) Scop.	LT: grabuzda, grobūzdai, grobūzdas	leaves	cooked	soup	4		1			
				fermented	cold soup			1			
	*Helichrysum arenarium* (L.) Moench; DZUPL049, DDZUPL13, DDZUPL28, DDZUPL30, DZULT134, DDZULT06, DDZULT37	LT: katpėdėlės, sausukai	aerial parts	dried	recreational tea					1	
			flowers	dried	recreational tea					1	
	*Matricaria chamomilla* L.; DDZUPL14, DDZUPL07, DZUPL026, DDZULT07, DDZULT26, DZULT036, DZULT059	PL: ramunek, rumianek, ramašačka, ramaška, ramonki, romashka, rumianki, rumiańki LT: ramunėlės, ramunėliai, laukiniai, ramunukai, ramunėlės, ramunukai, ramunės, rumianki, romashka, ramunukai, ramaška, rumianački	aerial parts	dried	recreational tea	11	7	2	5	6	
			flowers	dried	recreational tea	1		1	4	5	7
	*Taraxacum officinale* (L.) Weber ex F.H.Wigg.; DZUPL051, DZUPL064, DDZUPL09	PL: pienė, pienės LT: mlecz, mniszek, aduvančyk	flowers	fresh	recreational tea					1	
					salad					1	
					snack			1			
				cooked	syrup	1	3	2		2	2
			leaves	fresh	salad		1	3		2	1
	*Tussilago farfara* L.; DZUPL058, DZULT108	LT: podbieł	flowers	dried	recreational tea					1	
Berberidaceae	*Berberis vulgaris* L.	PL: barbarys	fruits	cooked	compote		2				
Betulaceae	*Alnus* spp. ( *Alnus incana* (L.) Moench, *Alnus glutinosa* (L.) Gaertn.)	PL: olcha, olsza, olszyna, alcha, alšyna, volcha, olšyna LT: alcha, alksnis, ankšliai, juodalksnis, ol’kha	wood	dried	to smoke meat and fish	3	8	11	9	20	18
	*Betula* spp. ( *Betula pendula* Roth, *Betula pubescens* Ehrh.); DZUPL053, DZULT013, DZULT050	PL: biaroza, brzoza, beržas, bereza, bžoza, biarjeza LT: beržas, bieroza, bieržalis, bereza, biržas, biaroza	buds	dried	recreational tea			2			
			leaves	dried	recreational tea			2			
			sap	fermented	kvass		1	1	1	2	
					drink	3	1	8	2	8	1
				fresh	drink	8	20	17	19	19	17
				frozen	drink					4	1
				processed	drink	8	18	5	8	1	1
			wood	dried	to smoke meat	1	3	1	2	2	1
	*Corylus avellana* L.; DZUPL078, DZULT127	PL: lazdynas, leszczyna, orzech, arešnik, arešyna, laščynaLT: lazdynas, riešutai, laščyna	seeds	dried	snack	4	2	20	5		14
			wood	dried	to smoke meat			1			
Boraginaceae	*Borago officinalis* L.; DZULT104, DZUPL021	LT: agurklė, aguročiai, ogurečnik	flowers	dried	recreational tea			1			
			leaves	fresh	salad			2		1	
			seeds	dried	recreational tea			1			
	*Symphytum officinale* L.; DZUPL046, DZUPL069, DZULT045, DZULT070	LT: riebešaknis, živakostas	leaves	fresh	salad			1			
Brassicaceae	*Armoracia rusticana* P.Gaertn., B.Mey. & Scherb.; DZULT022, DZUPL024	PL: chren, chrzan, krzan, chšanLT: chrienas, krienai, krienas, chren, kren	leaves	fresh	seasoning for lactofermented cucumbers	9	14	3	13	14	21
					seasoning for meat		1				2
					under bread during baking				1	2	3
			roots	fresh	seasoning for lactofermented cucumbers	1	8		3	17	17
					seasoning for meat dishes	8	21	4	13	27	21
	*Capsella bursa-pastoris* (L.) Medik.; DZULT024	LT: triskiautė žvakidė	aerial parts	dried	recreational tea			1			
	*Thlaspi arvense* L.; DDZUPL32	LT: bogužai, bogužis, bogužus	seeds	dried	seasoning					4	
Campanulaceae	*Campanula* sp.	LT: skambučai	flowers	fresh	snack					1	
Cannabaceae	*Humulus lupulus* L.; DZUPL009	PL: chmiel LT: apyniai	cones	dried	added to beer		2		1		1
					recreational tea			1			1
Caryophyllaceae	*Stellaria media* (L.) Vill.; DZULT099	PL: makryca LT: makryca, žliūgė	aerial parts	cooked	soup				2		
				fresh	salad	1		3			
Cupressaceae	*Juniperus communis* L.; DZUPL057, DZULT001	PL: aglis, kadagys, jadłowiec, jałowiec, jedłowiec, kadugys, jedłaviec, jełaviec, mažževielnik, jałaviec LT: ėglis, jėglis, jieglalis, kadagys, kadugys, ėglukas erškėtukas, jałaviec jadłaviec	fruits	dried	seasoning for meat and fish		1	1	1		5
					seasoning for sauerkraut					1	1
			wood	dried	to smoke meat and fish	3	10	13	12	17	6
Elaeagnaceae	*Elaeagnus rhamnoides* (L.) A.Nelson	PL: oblepicha LT: šaltalankis	fruits	fresh	drink			1			
					snack		1			1	
Equisetaceae	*Equisetum pratense* Ehrh.; DDZUPL10	LT: ožkabarzdis	aerial parts	dried	recreational tea					1	
Ericaceae	*Calluna vulgaris* (L.) Hull; DZULT126	LT: viržis	flowers	dried	recreational tea			1			
			seeds	dried	bread additive			2			
	*Empetrum nigrum* L.	LT: varnavuogės	fruits	cooked	compote	2					
					jam	4					
				fresh	snack	3					
				frozen	raw jam	2					
	*Vaccinium myrtillus* L.; DZUPL056, DZULT100	PL: czarne, czarne jagody, czarnicy, czarnicznik, mėlynės, čarnika, čarnicy, čarnička, chernaya yagoda, chernika, chernyye yagody, čornaja jahada, čornyja, čornyja jahady LT: čarnika, juodos, uogos, čarnyca, čarnykai, čarnicy, čornyja jahady, juodos, mėlynės, mėlynė, mėlyneuogės, mėlynos uogos, juodos uogos, čarničnik, chernika	aerial parts	dried	recreational tea	4	3	7	3		
			fruits	cooked	compote	5	7	3	4	3	1
					jam	18	25	21	13	22	12
				dried	recreational tea			1			
					snack	2	2	1	3		4
				fresh	additive to yogurt	1					
					dessert with milk (sugar)	2		2		3	2
					juice						1
					added to pies		1		3		2
					snack	9	12	3	12	4	17
				frozen	dumplings						1
					raw jam	4	3	4	3	3	1
					snack		2	2	2		
			leaves	dried	recreational tea			1			
	*Vaccinium oxycoccos* L.	PL: klukwa, spanguolės, żurawina, żurawiny, klyukva, žuraviny LT: spalgenos, spanguolės, žuraviny, klyukva	aerial parts	dried	recreational tea			1			
			fruits	cooked	compote			1			
					jam	1	1	2	2	4	6
				fresh	dessert with sugar			1		1	
					kissel	2		1	3	2	2
					added to pies				1		2
					seasoning for meat					4	2
					seasoning for sauerkraut	7	8	2	8	5	10
					snack	2		3	4	3	4
					taste additive to alcohol						3
				frozen	raw jam			2	1	2	
	*Vaccinium uliginosum* L.	PL: pjanicy LT: galubika, girtuoklės, buruvka, žaminės, uogos, sinitsa, sinicy	fruits	cooked	compote	1					
					jam	2					
				fresh	dessert					1	
					snack	4			1	2	
	*Vaccinium vitis-idaea* L.; DZUPL055	PL: borówka, borówki, bruknės, brusnicy, brusnika, boruvki, bruśnika, bruśnicy, bruśničnik LT: bruknė, bruknės, brukneuogės, bruknojai, brusnychnik, bruknienojai, brusnykai, juodos uogos, brusnika, bruśnika, bruśnica, bruśničnik	aerial parts	dried	recreational tea	1	1				
			fruits	cooked	compote	2	1	1		1	
					jam	5	1	17	9	5	5
				fresh	additive to yogurt	1					
					dessert			1		1	
					juice			1			
					kissel					1	
					added to pies				1		1
					seasoning for meat	1		1			
					seasoning for sauerkraut		1				
					snack	4	2	6	5	1	3
				frozen	raw jam	4		6	1	2	2
			leaves	dried	recreational tea	1		3	1		
Fabaceae	*Robinia pseudoacacia* L.	PL: akacja LT: akacija	flowers	cooked	jam						1
				fresh	dessert						1
			fruits	fresh	snack					1	
	*Trifolium pratense* L.; DZUPL068	PL: koniuczyna LT: dobilas, klever, raudoni dobilai	flowers	dried	recreational tea			3			
				fresh	snack	2					
					salad						1
Fagaceae	*Quercus robur* L.; DZULT048, DZUPL086	PL: dąb, dub LT: aožolas, ąžuolas, aržuolas, ąžuolas, ąžuolo žievė, dąb, dub, ūžuolas	acorns	roasted	coffee substitute			2	1		
				fresh	snack	1					
			bark	dried	taste additive to alcohol	1		1			
			leaves	dried	under bread during baking		1	4	5		
				fresh	seasoning for lactofermented cucumbers	1	2	2	2	6	7
			wood	dried	to smoke meat and fish	1	1	1		1	3
Hypericaceae	*Hypericum* spp. (*Hypericum maculatum* Crantz, *Hypericum perforatum* L.); DDZUPL08, DZUPL034, DZUPL087, DZUPL103, DDZULT20, DZULT075	PL: dziurawiec, jonažolės, zwieraboj, źvieraboj LT: jonažolės, jonažolinai, švento jankos, zvieraboj, svianty jansky, śvientajanskija ziołki	aerial parts	dried	recreational tea	6	2	7	3	1	1
Lamiaceae	*Leonurus cardiaca* L.; DZUPL085	LT: širdininkai	aerial parts	dried	recreational tea			1			
	*Melissa officinalis* L.; DZULT014, DZUPL037, DDZUPL18, DDZULT01	PL: melisa LT: melisa	leaves	dried	recreational tea	3	2	5	1	2	1
					taste additive to alcohol			1			
	*Mentha* spp. (*Mentha spicata* L., *Mentha × piperita* L.); DDZUPL04, DZUPL004, DZUPL007, DZUPL032, DZUPL047, DZUPL106, DDZULT11, DZULT021, DZULT043	PL: mėta, miata, mięta, miata miedzinaja, mielisa LT: karčioji mėta, pipirmėtė, mėta, mėta šokoladinė, mėtos, miata pieriecnaja, miata, miata pieračnaja, paprova miata	aerial parts	dried	recreational tea	9				1	1
					seasoning	1					
					seasoning for processed birch sap	1					
			leaves	dried	recreational tea	8	14	10	12	12	12
					seasoning for meat	2					3
					taste additive to alcohol			1			
	*Nepeta cataria* L.; DZULT076	LT: citrininė katažolė, melisa	leaves	dried	recreational tea	3		1			
					seasoning	1					
	*Origanum vulgare* L.; DZUPL063, DZUPL025, DDZUPL23, DDZUPL25	PL: macierzanka, macierzynka, dušyca LT: čobraliai, mociežanka	aerial parts	dried	recreational tea					1	1
					seasoning		1			3	1
					seasoning for blood soup					1	
					seasoning for cheese					1	
					seasoning for meat						3
	*Thymus* spp. (*Thymus pulegioides* L.); DZULT007, DZULT026, DZUPL039, DDZUPL19, DDZUPL31	PL: čiobreliai, czambor, czamborek, čabarok, čabrjelaj, chabrets, čambor, čamborek, čombar LT: čiobreliai, čiobrelis, čiombaras, čobraliai, tymianek, čabrec	aerial parts	dried	recreational tea	2	5	8	7	11	
					seasoning		1	1		1	
					seasoning for bread		1				
Malvaceae	*Tilia cordata* Mill.; DDZULT10, DDZULT14, DZULT031, DDZUPL02, DDZUPL29, DZUPL077	PL: liepžiedžiai, lipa LT: liepa, liepos žiedai, liepukai, liepžiedžiai, lipa	flowers	dried	recreational tea	5	12	13	4	8	7
			wood	dried	to smoke meat						1
Onagraceae	*Epilobium angustifolium* L.; DDZUPL16	PL: iwan-czaj, ivan-chay, ivan-čaj LT: gaurometis, ivan-chai	aerial parts	fermented	recreational tea		1				
			leaves	dried	recreational tea			2	1	2	
				fermented	recreational tea			1			
			roots	dried	drink		1				
	*Oenothera biennis* L.; DZULT118	LT: naktivaiša	flowers	fresh	snack			1			
Oxalidaceae	*Oxalis acetosella* L.	PL: zajęczy szczaw LT: kiškio kopūstai	leaves	fresh	snack	1				1	1
Papaveraceae	*Papaver* sp.	PL: mak LT: mak	seeds	cooked	dumplings	1					
					pastries		3				1
					pies	1	4				
Pinaceae	*Pinus sylvestris* L.; DDZULT02, DDZULT15, DZULT051, DZUPL073	PL: sasna LT: pušis	leaf buds	fresh	snack	2					
			shoots	fresh	snack					1	
			wood	dried	to smoke meat		1				
Plantaginaceae	*Plantago major* L.; DZULT004, DZUPL102	PL: babka lancetovata LT: gysločus, babka lancatavata	leaves	dried	recreational tea						1
				fresh	snack					1	
Poaceae	*Anthoxanthum nitens* (Weber) Y.Schouten & Veldkamp	LT: stumbražolė	aerial parts	dried	taste additive to alcohol			1			
Polygonaceae	*Rumex acetosa* L.; DZULT005, DZULT030, DZUPL084	PL: rūgštynės, rūškyniai, szczaujo, szczaw, szczawel, szczawuje, ščav, ščaviej, ščaviel, ščaŭ, ščaŭje, shchavel’, shchavl’ LT: rugštynės, ruškynės, rūgštynės, ruškyniai, rūštynės, ščaŭja, ščaviel, ščaŭje, shchavel’	leaves	cooked	cold soup			8			
					soup	31	37	35	23	38	28
				dried	soup			1			
				fermented	soup	1					
				fresh	salad	1	1				1
					snack	2				1	
				frozen	soup	1	3	2		5	7
				salted	soup	15	16	2	5	1	15
Rosaceae	*Crataegus* sp.; DZULT095	PL: bajaryšnik, głog LT: bajaryšnik, gudobelė	fruits	cooked	compote		3				
				dried	recreational tea	1				1	1
				fresh	snack		1			1	
				added to alcohol	alcoholic drink						1
			leaves	dried	recreational tea						1
	*Fragaria vesca* L.; DZULT025, DZULT037, DZUPL079	PL: czerwone, czyrwone, poziomki, žemuogės, zemlyanika, ziemlanika, krasnyje jahody, krasnyja, paziomki, čyrvone LT: pažiemkos, žamuogės, žamynavuogės, žemuogės, žemvuogės, žemyneuogės, zemlyanika, ziemlanika	aerial parts	dried	recreational tea			2	1	2	
			fruits	cooked	compote	5	2	1		1	
					jam	12	3	5	1	5	
				dried	recreational tea	1	1		1		
				fresh	additive to yogurt	1					
					dessert	3		2		3	
					drink					1	
					recreational tea		1				1
					snack	12	17	5	9	6	11
				frozen	raw jam	6	1	5		3	
					snack		1				
				added to alcohol	alcoholic drink						1
			leaves	dried	recreational tea	3	4	3	2	1	2
	*Malus sylvestris* (L.) Mill.	PL: jabłoń LT: laukinė obelis, laukiniai obuoliai	flowers	dried	recreational tea			1			
			fruits	dried	snack						1
				fresh	juice						1
					snack			1			
				frozen	snack			1			
	*Pyrus pyraster* (L.) Burgsd.	PL: grusza, hruša LT: kriaušė, laukinė kriaušė	flowers	dried	recreational tea			1			
			fruits	cooked	compote	1					4
				dried	snack		1		2		1
	*Rosa* sp.; DZUPL018, DZUPL061	PL: dzika róża, róża, szypownik, šypoŭnik, šypšyna LT: erškėtrožė, erškėtrožės, šypoŭnik, šypšyna, roza	flowers	dried	recreational tea	1	1				
			fruits	dried	recreational tea	3	3	1	1	1	4
				fresh	jam						1
					snack					1	
	*Rubus caesius* L.	PL: jeżyna, ježyna LT: ažiną, gervuogė, ažinykas, gervuogės, jažavika, ježavika	aerial parts	fermented	recreational tea			1			
			fruits	cooked	compote	2		1			
					jam	5	1	2		1	
				fermented	wine			1			
				fresh	additive to yoghurt	1					
					dessert			1			
					juice			1			
					snack	5	1	1		1	
				frozen	raw jam	1		2			
	*Rubus idaeus* L.; DZULT028, DZULT107, DZUPL054	PL: avietės, malina, maliny, krasnyja LT: avietė, avietės, malina, avytevuogės, avytevuogis, malinykas	stems	dried	recreational tea	2	2	1	1	2	1
					to smoke meat			1			
			fruits	cooked	compote	6	5	2	3	5	1
					jam	11	9	16	9	17	4
					kissel	1					
					syrup		1				
				fermented	wine					1	1
				fresh	additive to yoghurt	1	1				
					dessert with milk (sugar)	2		1	1	1	
					juice		1			5	
					added to pies						1
					added to alcohol			1			1
					snack	8	5	4	5	6	7
				frozen	raw jam	8	3	7	1	3	1
					snack		1		1		
			leaves	dried	recreational tea	3	1	1			3
				fermented	recreational tea			1			
	*Sorbus aucuparia* L.; DZULT009, DZUPL002	PL: jarzębina, šermukšnis, rabina LT: šermukšniai, šermukšnis	flowers	dried	recreational tea			2	1		
			fruits	cooked	jam				2	1	
					syrup			1			
				fresh	juice					1	
					recreational tea						1
					snack			4	1	3	
				frozen	raw jam					1	
					recreational tea				1		
					snack			1			
Salicaceae	*Populus tremula* L.	LT: drebulė, topolis	leaves	dried	under bread during baking	1					
			wood	dried	to smoke meat			1			
Santalaceae	*Viscum album* L.	LT: amalas	leaves	fresh	taste additive to alcohol	1					
Sapindaceae	*Acer platanoides* L.; DZULT029, DZULT062	PL: jawor, klevas, klon LT: klevas, klon, klianas	leaves	dried	under bread during baking		2	9	8		
				fresh	under bread during baking			4	6		
			sap	fermented	drink	1		1	2	1	
				cooked	syrup			1			
				fresh	drink	6	8	15	14	12	4
				frozen	drink					3	
			wood	dried	to smoke fish					1	
	*Aesculus hippocastanum* L.; DZULT034, DZULT057, DZUPL008	LT: kaštonas	seeds	roasted	coffee substitute			1			
Urticaceae	*Urtica dioica* L.; DZULT002, DZULT017, DZUPL083, DDZUPL01, DDZUPL07	PL: dilgėlės, dilginės, pokrzywa, krapiva, krapiŭka, pokšyva LT: dilgėlas, dirgėlė, dilgėlė, dilgėlės, dilginės, dirgėlės, notrės, dzirgėlė, dzilgėląs, dzirgėlės, krapiva	aerial parts	cooked	soup	1		3	1	3	
				dried	recreational tea					2	
					soup				1	1	
				fresh	drink					1	
					recreational tea					1	
					salad					2	
					to preserve fresh meat						2
			leaves	cooked	soup	7	15		10		5
				dried	recreational tea					1	2
					seasoning		1				
					soup		1				
				fresh	recreational tea						1
					salad		2				
					snack			1			
			seeds	cooked	soup					1	
			aerial parts in spring	cooked	soup	17	5	17	7	16	
					added to sandwiches			1			
				dried	recreational tea					9	
					soup			1			
				fresh	salad	1				4	1
	*Urtica urens* L.; DZULT053, DZUPL059	PL: pokrzywa LT: dirgalas, krapiva	leaves	cooked	soup	1					1
Viburnaceae	*Sambucus nigra* L.; DZULT081, DZUPL013, DZUPL016, DDZUPL07, DDZUPL15, DDZUPL22, DDZUPL27	PL: czarny bez LT: bezas, biały bez, juodas bezas, čarny bez, juodi bezai, šeivamedis	flowers	dried	recreational tea					3	1
				cooked	syrup					1	
					dessert					1	
			fruits	cooked	compote						1
					jam					1	
				dried	recreational tea					1	
				fresh	juice					1	
	*Viburnum opulus* L.; DZULT010	PL: kalina, putinas LT: kalina, putinas	flowers	dried	recreational tea				1		
			fruits	fresh	recreational tea				1		
					snack				1		
			fruits	cooked	compote	1					
					jam	1					
					syrup				1		
				dried	recreational tea			2			
				fresh	dessert with sugar			1			
					recreational tea			1			
					seasoning for sauerkraut	1		1			
					snack	1					
				frozen	raw jam			1	1		

**Table 3:** Influence of socio-demographic variables on the number of taxa mentioned by interviewees.

Variable	BYLT (*n* = 33)	BYPL (*n* = 36)	LTLT (*n* = 30)	LTPL (*n* = 37)	PLLT (*n* = 32)	PLPL (*n* = 32)	Test Total (*n* = 200)	*p*-Value Total (*n* = 200)
Gender (mean value of the number of taxa mentioned)	0–9.13 1–9 Student’s t: 0.127 *p* = 0.905	0–8.76 1–8.57 Student’s t: 0.089 *p* = 0.931	0–11.61 1–12.29 Student’s t: 0.248 *p* = 0.810	0–8.33 1–6.10 Student’s t: 2.093 *p* = 0.047	0–12.52 1–5.0 Student’s t: 4.621 *p* = 0.000	0–11.29 1–7.82 Student’s t: 2.469 *p* = 0.022	χ2 test: 8.45	0.133
Education level (mean value of the num-ber of taxa mentioned)	0–10 1–8 2–9.50 3–17 4–8.86 5–9.80	1–7.67 2–9 3–12.50 4–9 5–7 ANOVA: 1.048 *p* = 0.399	1–14.29 2–10 3–13.60 4–10.69 5–6.0	1–8.33 2–8 3–6.86 4–7.50 ANOVA: 0.251 *p* = 0.860	1–13 2–8.45 3–10.14 4–8.60 5–19 ANOVA: 3.854 *p* = 0.013	1–9 2–11.17 3–8.25 4–10.31 5–17	ANOVA: 1.159	0.331
Language (mean value of the num-ber of taxa mentioned)	1–10 2–9.63 3–9.10 4–5.67	1–7.25 2–8.41 3–10 ANOVA: 0.687 *p* = 0.510	1–5 2–19.50 3–10.95 4–11.40 ANOVA: 4.376 *p* = 0.013	1–7.27 2–7.52 3–8.67 4–11.0 ANOVA: 0.656 *p* = 0.585	2–10.68 3–11.30 Student’s t: 0.304 *p* = 0.304	1–9.97 2–11.33 Student’s t: 0.604 *p* = 0.595	ANOVA: 0.800	0.495

**Table 4:** The relative frequency of citation of the top 20 wild plants mentioned by interviewees in the study area.

Taxa	BYLT	BYPL	LTLT	LTPL	PLLT	PLPL
*Vaccinium myrtillus*	**0.647**	**0.829**	**0.800**	**0.684**	**0.688**	**0.844**
*Rubus idaeus*	**0.500**	**0.400**	**0.633**	**0.368**	**0.656**	**0.375**
*Rumex acetosa*	**0.853**	**0.800**	**0.833**	**0.500**	**0.781**	**0.844**
*Carum carvi*	**0.588**	**0.457**	**0.633**	**0.553**	**0.750**	**0.531**
*Urtica dioica*	**0.735**	**0.571**	**0.667**	**0.474**	**0.781**	**0.281**
*Betula* spp.	**0.441**	**0.686**	**0.567**	**0.632**	**0.656**	**0.594**
*Fragaria vesca*	**0.559**	**0.657**	**0.433**	**0.237**	**0.406**	**0.375**
*Armoracia rusticana*	**0.382**	**0.686**	**0.133**	**0.474**	**0.719**	**0.906**
*Vaccinium oxycoccos*	**0.324**	**0.229**	**0.200**	**0.368**	**0.281**	**0.594**
*Acer platanoides*	**0.176**	**0.257**	**0.733**	**0.579**	**0.375**	**0.125**
*Vaccinium vitis-idaea*	**0.235**	**0.143**	**0.600**	**0.316**	**0.219**	**0.281**
*Mentha* spp.	**0.529**	**0.371**	**0.300**	**0.316**	**0.344**	**0.438**
*Acorus calamus*	**0.235**	**0.086**	**0.500**	**0.158**	**0.469**	**0.625**
*Juniperus communis*	**0.088**	**0.314**	**0.400**	**0.342**	**0.500**	**0.312**
*Alnus* spp.	**0.059**	**0.229**	**0.333**	**0.237**	**0.469**	**0.562**
*Matricaria chamomilla*	**0.353**	**0.200**	**0.100**	**0.237**	**0.281**	**0.219**
*Tilia cordata*	**0.147**	**0.314**	**0.400**	**0.105**	**0.219**	**0.250**
*Quercus robur*	**0.118**	**0.114**	**0.333**	**0.132**	**0.188**	**0.312**
*Corylus avellana*	**0.088**	**0.057**	**0.433**	**0.105**	**0.000**	**0.406**
*Thymus* spp.	**0.059**	**0.143**	**0.233**	**0.184**	**0.344**	**0.000**

**Table 5:** Informant consensus factor.

Case Study	Sum of Use Reports (UR)	Number of Taxa Mentioned	Informant Consensus Factor (ICF)
BYLT	396	44	0.891139241
BYPL	359	37	0.899441341
LTLT	445	51	0.887387387
LTPL	339	33	0.905325444
PLLT	434	44	0.900692841
PLPL	371	41	0.891891892
			
Belarus	755	50	0.935013263
Lithuania	784	53	0.933588761
Poland	805	55	0.932835821
			
Poles	1275	53	0.959183673
Lithuanians	1069	55	0.949438202
			
All case studies	2344	72	0.96969697

**Figure A1:**
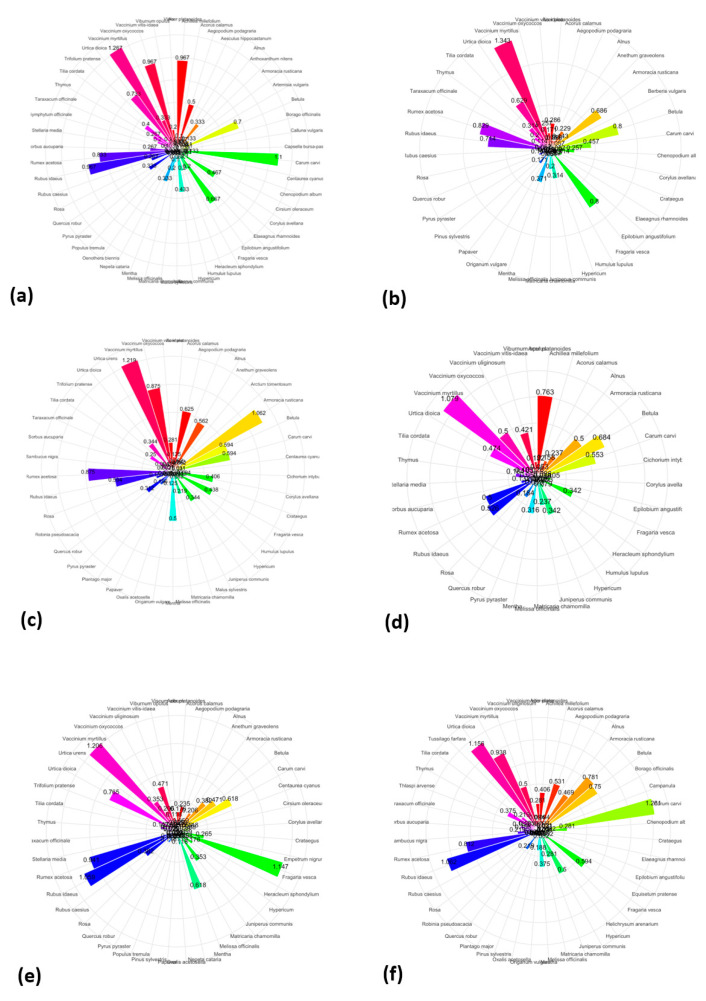
Radial plots of cultural importance (CI) index values for the case studies: (**a**) BYLT, (**b**) BYPL, (**c**) LTLT, (**d**) LTPL, (**e**) PLLT, and (**f**) PLPL. Analysis performed using EthnobotanyR package in RStudio.

## Data Availability

Data are available upon request to the corresponding author.

## Abbreviations

LEK—Local Ecological Knowledge; PL—Polish community; LT—Lithuanian community; BYLT—Lithuanians living in Belarus; BYPL—Poles from Belarus; LTLT—Lithuanians from Lithuania; LTPL—Poles living in Lithuania; PLLT—Lithuanians from Poland; PLPL—Poles living in Poland; UR—Use Reports; NU—Number of Uses; FL—Fidelity Level, RFC—Relative Frequency of Citation; ICF—Informant Consensus Factor; CI—Cultural importance; WWII—World War II.

## Appendix A

**Table A1:** Overview of ethnobotanical calculations.

Calculation	Formula	Explanation	Reference
Use reports (UR)	$URs=\overset{uNC}{\underset{u=u_{1}}{\sum }}\overset{iN}{\underset{i=i_{1}}{\sum }}UR_{ui}$	The total uses of the species by all interviewees within each use category for that species.	[58]
Number of uses (NU)	$NU_{S}=\overset{uNC}{\underset{u=u_{1}}{\sum }}$	The total number of use categories.	[58]
Fidelity level (FL)	$FLs=\frac{\left(Ns*100\right)}{FC_{s}}$	The percentage of interviewees who use a plant for the same purpose compared to all uses of the plant for any purpose, where *Ns* is the number of interviewees that use a particular plant for a specific purpose, and *FCs* is the frequency of citation for the species.	[59]
Relative frequency of citation (RFC)	$RFCs=\frac{FCs}{N}=\frac{\sum_{i=i1}^{iN}URi}{N}$	The frequency of citation for each species *s*, where *URi* refers to the use reports for all interviewees *i*, and *N* is the total number of interviewees in the survey.	[60]
Cultural importance (CI)	$CIs=\overset{uNC}{\underset{u=u_{1}}{\sum }}\overset{iN}{\underset{i=i_{1}}{\sum }}UR_{\frac{ui}{N}}$	The sum of the proportion of interviewees that mention the use of each species.	[59]
Informant consensus factor (ICF)	$ICF=\frac{Nur-Nt}{Nur-1}$	Quantitative parameter to evaluate of agreement among interviewees’ knowledge circulated, where *Nur* is the number of use reports in the food category, and *Nt* is the number of species (taxa).	[61]
